# Predicting survival in glioblastoma with multimodal neuroimaging and machine learning

**DOI:** 10.1007/s11060-023-04439-8

**Published:** 2023-09-05

**Authors:** Patrick H. Luckett, Michael Olufawo, Bidhan Lamichhane, Ki Yun Park, Donna Dierker, Gabriel Trevino Verastegui, Peter Yang, Albert H. Kim, Milan G. Chheda, Abraham Z. Snyder, Joshua S. Shimony, Eric C. Leuthardt

**Affiliations:** 1 Department of Neurological Surgery, Washington University School of Medicine, St. Louis, MO 63110 USA; 2 Center for Health Sciences, Oklahoma State University, Tulsa, OK 74136 USA; 3 Department of Neuroscience, Washington University School of Medicine, St. Louis, MO 63110 USA; 4 Mallinckrodt Institute of Radiology, Washington University School of Medicine, St. Louis, MO USA; 5 Brain Tumor Center at Siteman Cancer Center, Washington University School of Medicine, St. Louis, MO USA; 6 Department of Medicine, Washington University School of Medicine, St. Louis, MO USA; 7 Department of Neurology, Washington University School of Medicine, St. Louis, MO USA; 8 Department of Biomedical Engineering, Washington University in Saint Louis, St. Louis, MO 63130 USA; 9 Department of Mechanical Engineering and Materials Science, Washington University in Saint Louis, St. Louis, MO 63130 USA; 10 Center for Innovation in Neuroscience and Technology, Washington University School of Medicine, St. Louis, MO 63110 USA; 11 Brain Laser Center, Washington University School of Medicine, St. Louis, MO 63110 USA; 12 National Center for Adaptive Neurotechnologies, Albany, USA

**Keywords:** Deep learning, Glioblastoma, Cortical thickness, Functional MRI, Survival

## Abstract

**Purpose:**

Glioblastoma (GBM) is the most common and aggressive malignant glioma, with an overall median survival of less than two years. The ability to predict survival before treatment in GBM patients would lead to improved disease management, clinical trial enrollment, and patient care.

**Methods:**

GBM patients (N = 133, mean age 60.8 years, median survival 14.1 months, 57.9% male) were retrospectively recruited from the neurosurgery brain tumor service at Washington University Medical Center. All patients completed structural neuroimaging and resting state functional MRI (RS-fMRI) before surgery. Demographics, measures of cortical thickness (CT), and resting state functional network connectivity (FC) were used to train a deep neural network to classify patients based on survival (< 1y, 1-2y, >2y). Permutation feature importance identified the strongest predictors of survival based on the trained models.

**Results:**

The models achieved a combined cross-validation and hold out accuracy of 90.6% in classifying survival (< 1y, 1-2y, >2y). The strongest demographic predictors were age at diagnosis and sex. The strongest CT predictors of survival included the superior temporal sulcus, parahippocampal gyrus, pericalcarine, pars triangularis, and middle temporal regions. The strongest FC features primarily involved dorsal and inferior somatomotor, visual, and cingulo-opercular networks.

**Conclusion:**

We demonstrate that machine learning can accurately classify survival in GBM patients based on multimodal neuroimaging before any surgical or medical intervention. These results were achieved without information regarding presentation symptoms, treatments, postsurgical outcomes, or tumor genomic information. Our results suggest GBMs have a global effect on the brain’s structural and functional organization, which is predictive of survival.

**Supplementary Information:**

The online version contains supplementary material available at 10.1007/s11060-023-04439-8.

## Introduction

Glioblastoma (GBM) is the most common and deadly primary tumor of the central nervous system, with an age-adjusted incidence rate of 3.22 per 100,000, accounting for approximately 70% of newly diagnosed cases [1, 2]. Further, studies have indicated an increase in the incidence rates of GBM [3, 4], and within certain populations, specifically the elderly, the mortality rate has also increased [5–7]. The standard first-line treatment for newly diagnosed GBM is surgical resection and radiotherapy with concomitant temozolomide, followed by adjuvant temozolomide [8, 9]. Despite advances in stratified treatment, the median overall survival (OS) rate and progression free survival rate remains at approximately 14 months and 7 months respectively, with only 43% of patients surviving one year and 6.9% surviving five years post-diagnosis [1, 10, 11]. Intertumor/intratumor heterogeneity, characterized by distinct genetic alterations, rapid proliferation, aggressive infiltration, multiple activated signal transduction pathways, and the emergence of treatment-resistant cells soon after therapy onset, limits the effectiveness of current therapies, leading to inevitable tumor recurrence and death [8, 12]. Given the dismal survival statistics and the heterogeneity of the disease, a non-invasive method that reliably predicts a patient’s OS would benefit clinicians, patients, and their families.

Preoperative MRI is necessary for determining the best approach to surgical resection. To optimize patient outcomes, the surgeon must weigh the extent of resection against its impact on functional preservation [13, 14]. This equates to finding the optimal balance between the extent of resection, quality of life, and survival [15, 16]. Structural MRI (e.g., T1-weighted pre- and post-contrast, T2-weighted, and diffusion tensor imaging) is routinely employed to evaluate the location and size of the tumor, as well as morphological changes in brain anatomy distant from the tumor. Structural MRI is crucial to planning the extent of resection. Further, structural measures, such as cortical thickness, have been identified as global markers of severity in brain disease. This has been described across a spectrum of brain disorders, including dementia (Alzheimer’s and Parkinson’s disease), metabolic disorders (e.g. anorexia nervosa), and psychiatric disorders (e.g. major depression and schizophrenia) [17–20]. Recently, it has also been shown to be a strong predictive biomarker of survival in high grade glioma [21]. Similarly, functional MRI, i.e., blood oxygen level-dependent (BOLD) task or resting state fMRI (RS-fMRI) has been used in numerous studies to evaluate functional alterations that occur in neurodegenerative diseases [22–24]. In the context of brain tumors, RS-fMRI has shown promise as a biomarker of survival and as a method for mapping functional networks before resection to maximize functional preservation [25, 26].

Survival analysis has traditionally relied on methods such as the Kaplan-Meier estimator and the Cox proportional hazards model to predict outcomes in diseases like GBM. While these methods have been instrumental in understanding survival rates and associated factors, they often face challenges when dealing with high-dimensional data or intricate non-linear relationships inherent in complex diseases. Machine learning (ML) is a branch of artificial intelligence that builds models by extracting patterns from raw data [27]. ML-based survival prediction diverges from these traditional statistical methodologies. Specifically, while traditional methods rely on assumptions about underlying data distributions and hazard functions, ML techniques are data-driven, enabling them to adapt and model complex nonlinear relationships in the data without predefined constraints. ML has significant applicability to brain tumors, primarily in the context of image segmentation, which can classify brain tissue from MRI into multiple categories [16, 28, 29]. Beyond image segmentation, numerous studies have demonstrated the ability to infer clinically relevant information based solely on the radiomic features of MRI, such as predicting the tumor’s genetic characteristics, differentiation between pseudo and true progression, and classification of transcriptome-based subtypes [30–33]. Given the predictive capability of ML and the rich information embedded within radiomic features, we hypothesize that models trained on MRI data could be used to predict survival in GBM patients.

This research aims to use ML and multimodal neuroimaging to develop models capable of classifying three groups of overall survival in GBM patients. Deep feed-forward artificial neural networks (ANN) were trained to classify GBM patients (n = 133) into less than one year, one to two years, or greater than two years of survival (< 1y, 1-2y, >2y). Input to the models included demographics (age and sex), contralesional cortical thickness (CT), and resting-state network functional connectivity (FC) correlations derived from 15 resting-state networks, all calculated on preoperative data. The present objective of our study is to assess the prognostic value of these preoperative imaging features when considered in isolation, and not to construct a prognostic index that takes into account other clinical and demographic factors (outside of age/sex) which may hold independent prognostic significance. Our multimodal models can provide reliable and accurate survival classification without drawing upon other clinical variables such as genetics or the extent of resection. The primary strength of our approach lies in leveraging functional connectivity and cortical thickness to discern clinically pertinent insights about a patient’s mortality risk. We believe that this technology holds potential to enhance surgical planning, stratified therapy, and facilitate shared decision-making for GBM patients.

## Methods

### Patients

Patients with GBM (N = 133) were retrospectively recruited from the neurosurgery brain tumor service at Washington University Medical Center. All subjects were diagnosed with Glioblastoma on pathological examination of biopsy and resection acquired brain samples at the Division of Neuropathology between May 2012 and September 2020. Definitive diagnosis was achieved based on the presence of histomorphological and immunohistochemical characteristics supportive of glioblastoma using the appropriate WHO 2007 and 2016 guidelines [34, 35]. These findings include the presence of tumor cells with astrocytic-like appearance, microvascular proliferation, palisading necrosis, pleomorphic hyperchromatic nuclei, and frequent mitoses. Our cohort consists of 131 patients with the Diagnosis of GBM IDH wildtype and 2 patients with GBM IDH mutant. Under the recent WHO 2021 guidelines [36], the 2 IDH mutant patients would be classified as Grade IV Astrocytoma, IDH mutant based on the advanced role of molecular diagnostics in CNS tumor taxonomy. Inclusion criteria included a new diagnosis of brain tumor (first occurrence), biopsy or surgical treatment, and the availability of pre-surgical structural and functional MRI. Exclusion criteria included patients younger than age 18 and patients that were lost to follow-up. This study was approved by the Washington University in St. Louis Institutional Review Board.

### Clinical characteristics

Participant demographics and clinical characteristics are listed in Supplementary Table 1. Demographics included age and sex. Clinical characteristics include the overall survival, calculated as the difference between the first MRI and the date of death. Other clinical characteristics included the extent of resection (gross total (GTR), subtotal resection (STR), and near total resection (NTR), tumor location (frontal, parietal, temporal, occipital, cingulate, and/or other), Karnofsky Performance Status [37] (KPS > 70), O6-Methylguanine-DNA methyltransferase [38] (MGMT) promoter methylation status, epidermal growth factor receptor [39] (EGFR) amplification status, telomerase reverse transcriptase [40] (TERT) mutation status, isocitrate dehydrogenase 1 [41] (IDH1) mutation status, and phosphatase and tensin homolog [42] (PTEN) mutation status. We extensively collected the detailed history of medical comorbidities (Hx) that each patient presented with at the time of GBM diagnosis, including alcohol use disorder, tobacco use, hypertension, hyperlipidemia, chronic kidney disease (CKD), cardiac disease (myocardial infarction, arrhythmia, valvular dysfunction), deep vein thrombosis or pulmonary embolism (DVT/PE), psychiatric disorders, visual deficits, stroke, weakness, obesity (BMI > 30), diabetes, and headaches (migraine, tension, or cluster). Furthermore, we determined the presenting symptoms (Pw) of each patient, including weakness, visual changes, aphasia, hydrocephalus, confusion, headache, memory impairment, and seizures. Treatment regimens included patients treated with the Stupp protocol [43] (60 Gy radiotherapy plus concurrent and adjuvant temozolomide) and various clinical trials of radiotherapy, bevacizumab, CCNU, disulfiram, doxorubicin, and temozolomide (see Supplementary Table 1). Genetic data were measured by the Foundation Medicine commercial laboratory (https://www.foundationmedicine.com/) and the Washington University Genomics and Pathology service.

### MRI acquisition

All neuroimaging was performed on a Siemens Trio or Skyra 3T MRI scanner. Structural images included T1-weighted (T1w) magnetization prepared rapid acquisition gradient echo (MPRAGE: TE = 2.53 ms, TR = 1900 ms, TI = 900 ms, 256 × 256 acquisition matrix, 0.976 × 0.976 × 1 mm voxels), fluid-attenuated inversion recovery (FLAIR: 2D, slice thickness 5 mm, gap 1 mm, 256 × 256 matrix 0.9 × 0.9 mm pixel size, TE = 129 ms, TR = 8500 ms, TI = 2440 ms, flip angle 130), and T2-weighted (T2w) fast spin-echo (FSE: TE = 93 ms, TR = 5600 ms, 256 × 256 acquisition matrix, 1.093 × 1.093 × 2 mm voxels). RS-fMRI was acquired using a BOLD-sensitive EPI sequence (voxel size 3 mm^3^ isotropic; echo time = 27 ms; repetition time = 2.2–2.9 s; field of view = 256 mm; flip angle = 90). Two RS-fMRI runs were obtained in each patient yielding approximately 320 frames.

### MRI processing

Structural data preprocessing was performed with FreeSurfer (http://surfer.nmr.mgh.harvard.edu). Visual inspection of the segmentation results was performed for quality assurance purposes. In short, T1w and T2w images were visually inspected to ensure brain structures were free of blurring, ringing, striping, ghosting, etc., caused by head motion. Three raters (B.L., D.D., and G.V.) reviewed the segmentation to ensure data quality [21]. FreeSurfer-defined cortical parcels based on the Desikan-Killiany atlas [44] were used to define regional CT measures in the contralesional side of GBM patients. Preprocessing of fMRI data followed previously described methods and is detailed in the supplementary material (1.1).

Automated tumor segmentation was performed with a pre-trained convolutional neural network architecture [45] using post-contrast T1w, T2w, and FLAIR scans. The whole tumor mask was used for masking during atlas registration. The tumor segmentation maps were also used to create voxelwise heat maps showing the frequency of a voxel overlapping with the tumor segmentation. These methods are described further in the supplementary material (1.2).

### Functional connectivity

Regions of interest (ROI) were used to generate similarity maps for 15 resting state networks (RSN) based on previously published results [46]. ROIs were developed by taking each network’s top 200 probabilities (corresponding to the top 200 voxels). The networks include dorsal somatomotor (SMD), inferior somatomotor (SMI), cinguloopercular (CON), auditory (AUD), default mode (DMN), parietal memory (PMN), visual (VIS), frontoparietal (FPN), salience (SAL), ventral attention (VAN), dorsal attention (DAN), medial temporal (MET), reward (REW), thalamus (THA), and basal ganglia (BGA). The similarity between networks was calculated by computing the distance correlation [47] between the network-specific ROIs, resulting in 120 within and between network similarity measures. Because distance correlation first computes the distance matrix for each set of vectors, a similarity measure can be generated with a single calculation regardless of the spatial dimension of the two vectors. Thus, between-network correlations were calculated by comparing all 200 voxels for the two given networks in a single calculation and by averaging the distance correlation between each voxel and the other 199 voxels for within-network connections.

### Machine learning and statistical analysis

Analyses were performed in either MATLAB R2021b or R 4.2.1. Supplementary material (1.3) provides an in-depth description of the methods described in this section. In short, survival prediction was achieved using deep feedforward artificial neural networks (ANN) consisting of 3 hidden layers with eight neurons in each layer (Supplementary Fig. 1a). The model was trained to classify patients into less than one year, between one and two years, or greater than two years of survival. Input to the ANNs included age, sex, contralesional CT measures [21], and FC features. Dimensionality reduction was performed on the 120 FC features using an autoencoder with a single hidden layer consisting of 20 neurons (Supplementary Fig. 1b). A total of 11 encoded FC features (after removing nine sparse features) were used for training and are hereafter referred to as FC1, FC2,…FC11.

**Fig. 1:**
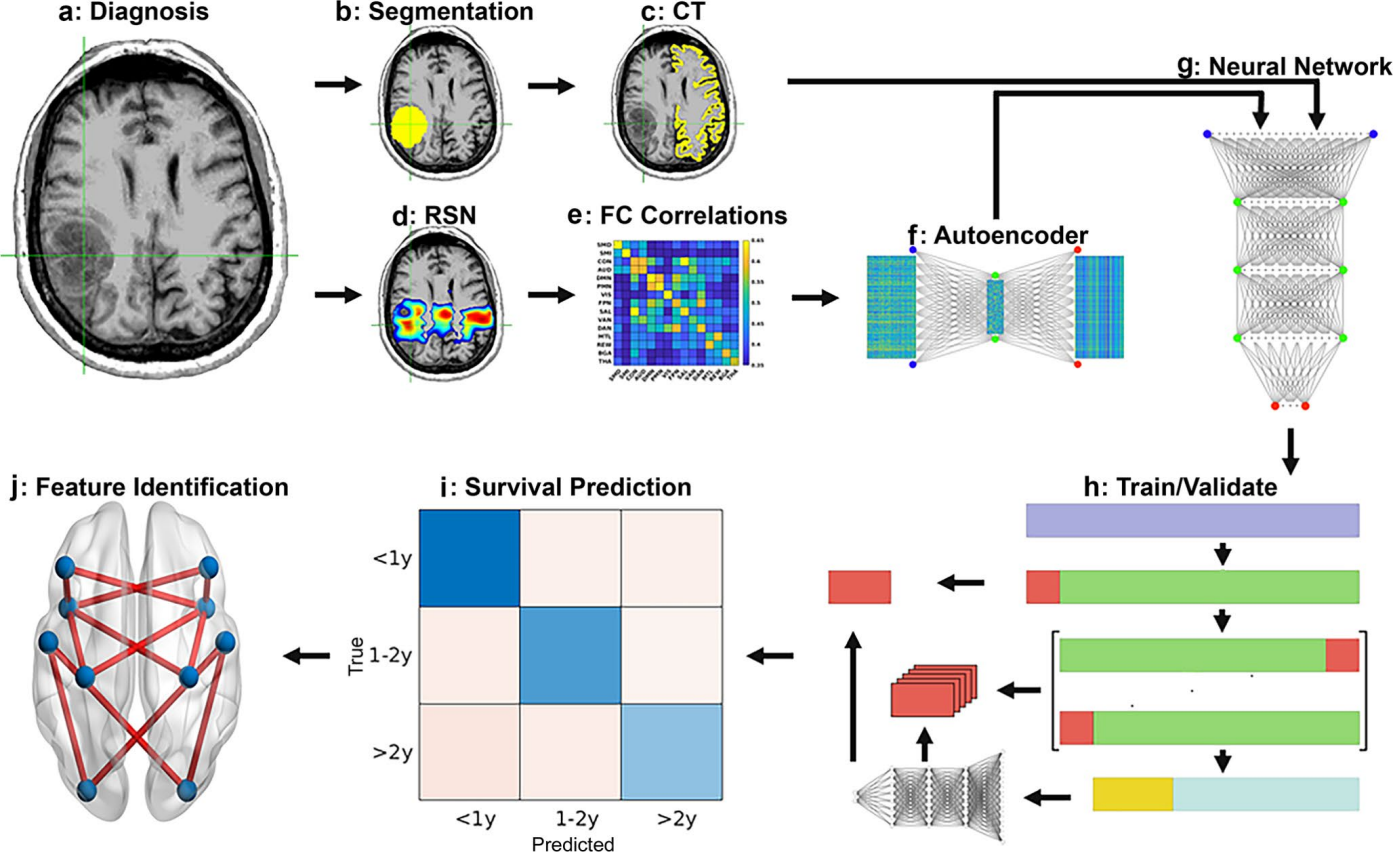
Survival prediction pipeline. Structural and functional MRI are collected at the time of diagnosis **(a)**. The tumor is then segmented **(b)**, and the segmentation is used for registration and identification of contralesional cortical thickness **(c)**. Resting state network ROIs **(d)** are partitioned and correlated **(e)**, and the RSN correlations are fed into an autoencoder **(f)** for dimensionality reduction. The contralesional CT and encoded FC features are combined and fed into a feedforward neural network **(g)**, trained with nested stratified cross-validation with global holdout **(h)**. Once the models are trained and validated, they are used to predict survival category **(i)**, and model-based features are identified **(j)** for presurgical planning

The procedure for the survival model’s training and validation involved nested stratified cross-validation and a hold-out approach. Further details on this process are provided in supplementary material Sect. 1.3 and supplementary Fig. 2. In contrast, the autoencoder was trained on 80% of the data, with the remaining 20% reserved specifically for validation termination. Permutation feature importance [48] was used to identify the strongest predictive features of survival based on the trained models and is described in detail in the supplementary material. Further, an average per-network feature weight was generated by averaging all within and between-network feature weights for each given network (e.g., to calculate the average feature weight for SMD, we averaged all feature weights associated with SMD [SMDxSMD, SMDxSMI, SMDxCON….]). Lastly, voxelwise FC feature maps were calculated by taking the dot product of the average feature weights with publically available FC probability maps [46]. The survival prediction pipeline and methods described herein are summarized in Fig. 1.

## Results

**Fig. 2:**
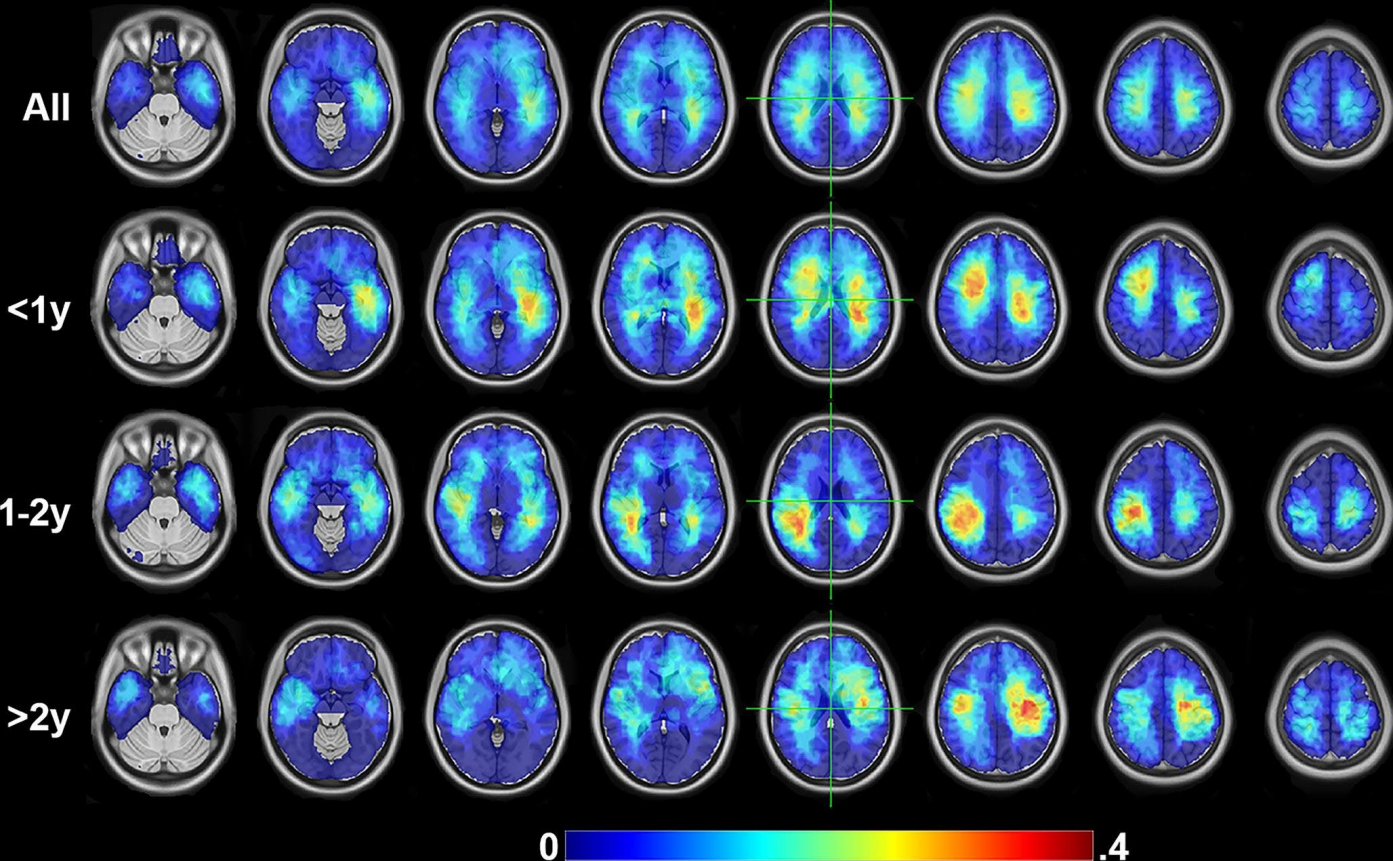
Spatial tumor frequency. Brain regions most affected by the tumors were calculated by averaging the tumor segmentation maps for all data and partitioned based on the survival group

Supplementary Table 1 provides information regarding demographics, median overall survival, tumor location, Karnofsky Performance Scale (KPS), treatments, genetic alterations, medical history, and presentation symptoms at the time of diagnosis in total and based on the three survival groups. The majority of the cohort was male (58%), with a mean age at diagnosis of 60.8 years and median overall survival of 14.1 months. Of the measures considered, ratios of sex (p = 0.01), KPS > 70 (p = 0.01), gross total resection (GTR, p < 0.01), full Stupp protocol (p < 0.01), bevacizumab (p < 0.01), CCNU (p = 0.01), doxorubicin treatment (p < 0.01), temozolomide treatment (p < 0.01), MGMT promoter methylation status (p = 0.03), and history of tobacco (p = 0.03) use were significantly different amongst survival groups (Chi-squared test [49]). Furthermore, patients presenting with symptoms of visual changes (p = 0.02), memory impairment (p = 0.04), and seizures (p = 0.01) showed significant differences among the survival groups (Chi-squared test). Supplementary Fig. 3 shows bar graphs for these variables. Age at diagnosis also significantly differed between groups (Kruskal-Wallis test [50], p = 0.04). Figure 2 shows the regions most affected by the tumors, calculated by averaging the tumor segmentation maps.

**Fig. 3:**
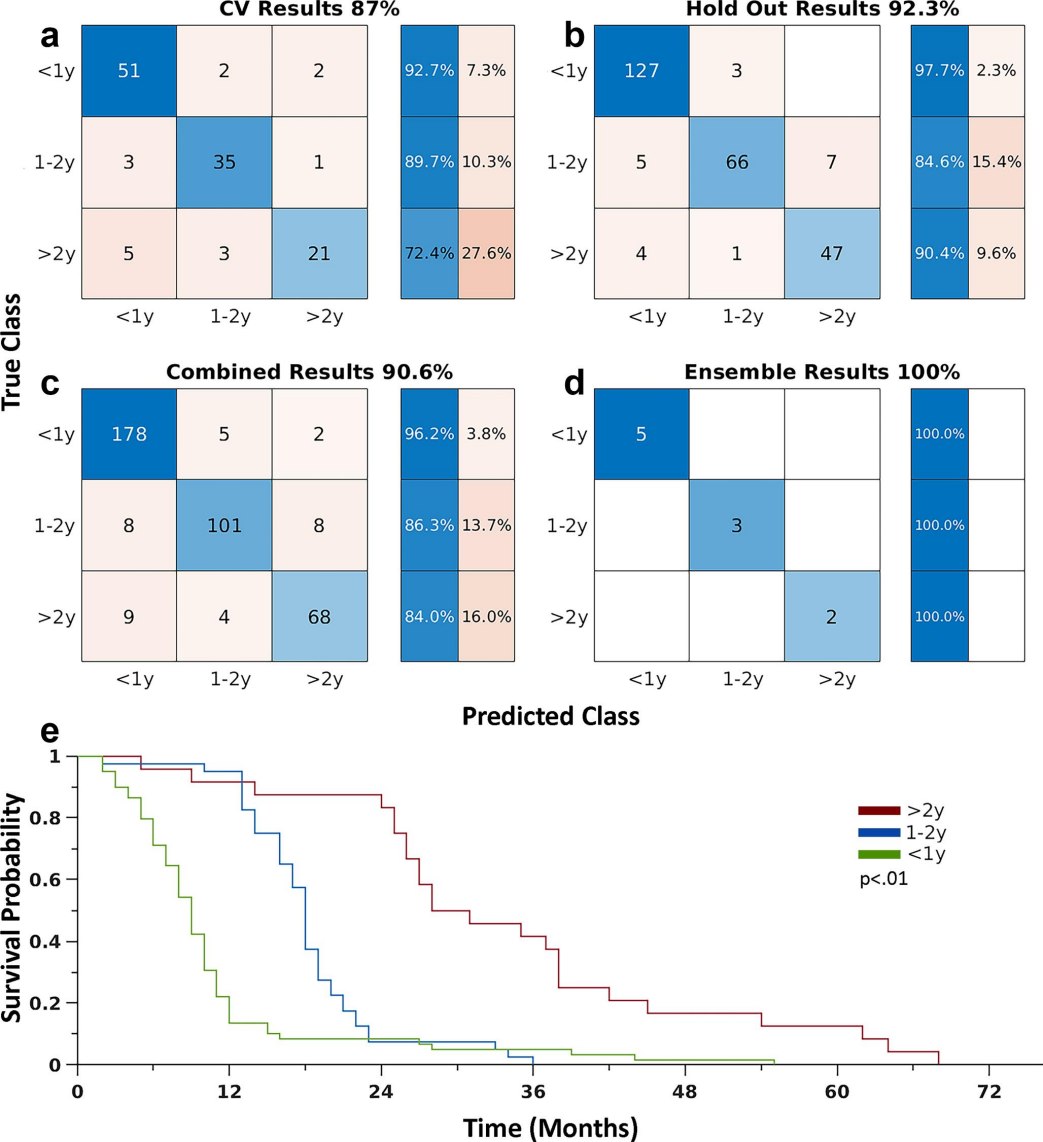
Model results. **(a)** The models achieved 87% cross-validation accuracy. **(b)** The total accuracy in testing the models on the ten held out samples was 92.3%. **(c)** Combination of the cross-validation and hold out results yields a total accuracy of 90.6%. **(d)** When treating all models as an ensemble and averaging the results on the ten held out samples, the model achieved 100% accuracy. **(e)** Kaplan-Meier survival curves based on model classification

Figure 3 shows the model results in classifying patients into the appropriate survival group. Overall, the models achieved 87% cross-validation accuracy (Fig. 3a). The total accuracy in testing the models on the ten held-out samples was 92.3% (Fig. 3b). When combining the cross-validation and hold-out results, the total accuracy was 90.6% (Fig. 3c). When treating all models as an ensemble and averaging the results on the ten held-out samples, the model achieved 100% accuracy (Fig. 3d). Figure 3e shows the Kaplan-Meier survival curves based on the model classifications of the cross-validation data (p < 0.01). Further, when patients are further segmented by the extent of resection and completion of Stupp protocol, the model still significantly separated patients’ survival. (Supplementary Fig. 4).

**Fig. 4:**
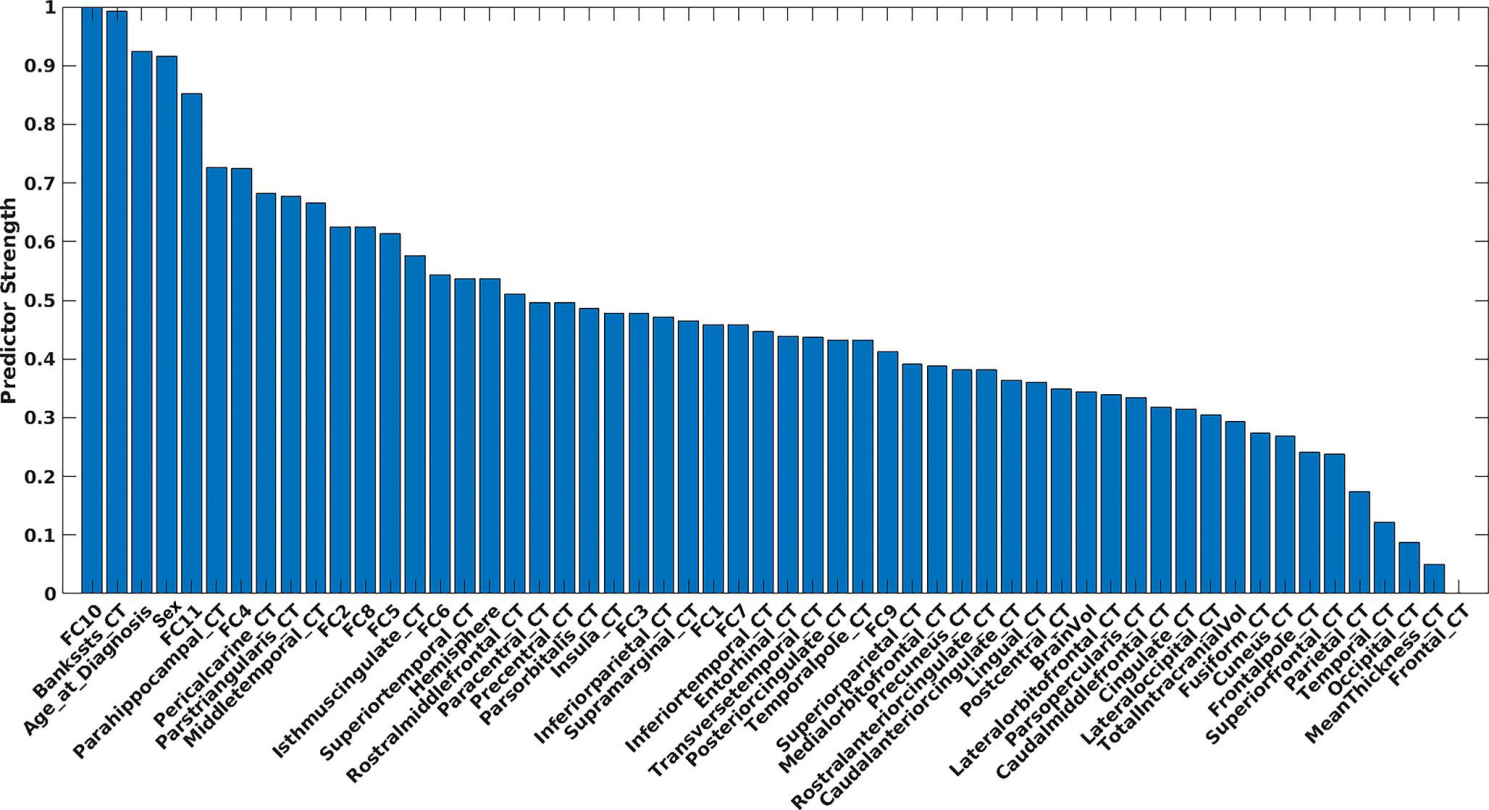
Strongest predictive features of survival. Age at diagnosis and sex were strong demographic predictors. Contralesional cortical thickness in the superior temporal sulcus, parahippocampus, pericalcerine, pars triangularis, and middle temporal regions was found to be strong predictors. Several encoded FC features were also strong predictors (FC10, FC11, and FC4)

**Fig. 5:**
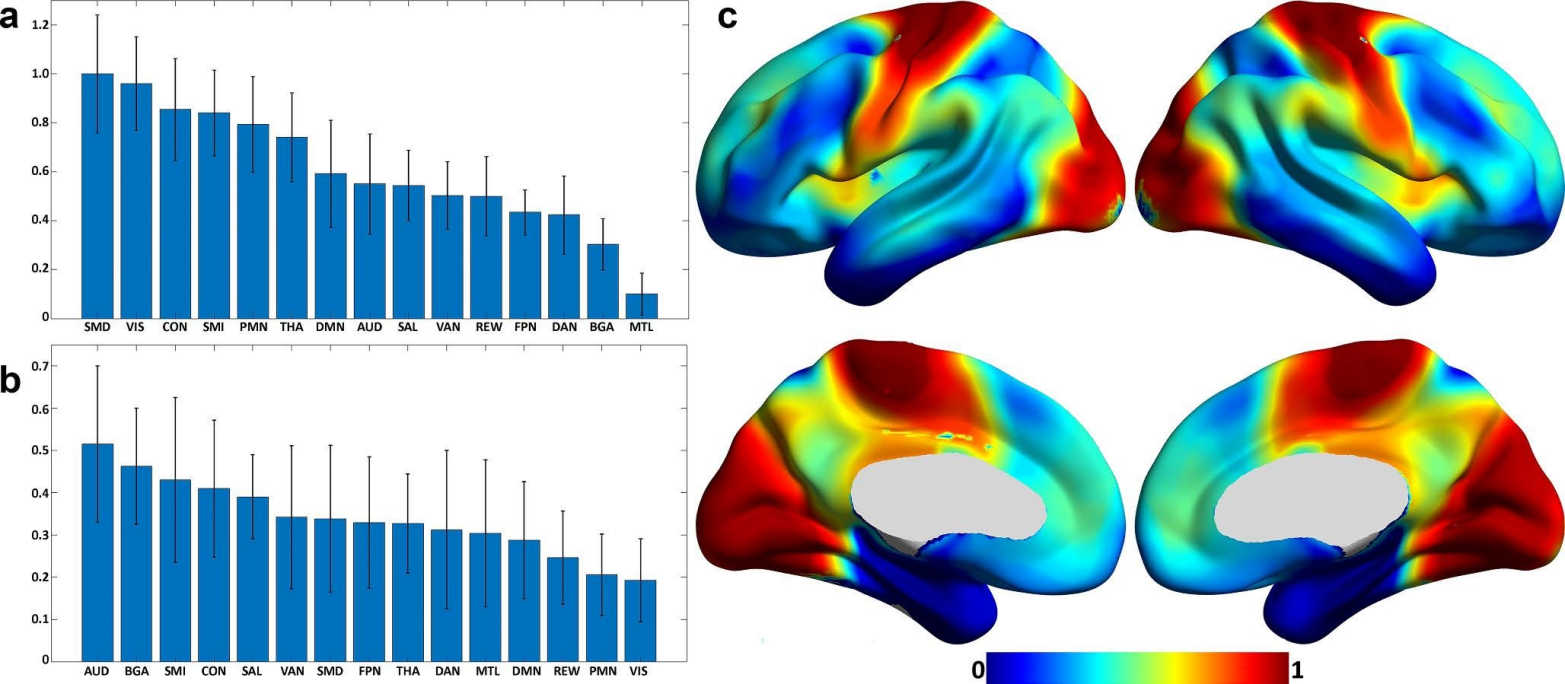
**(a)** Mean and STD of per-network feature weights calculated by averaging the within and between network feature weights for each network. SMD, VIS, CON, and SMI were the strongest average predictors. **(b)** Mean and STD of the voxelwise tumor frequency (Fig. 2) segmented based on published RSN segmentations. The networks with the strongest tumor frequency overlap included AUD, BGA, CON, and SMI. **(c)** Results of mapping the per-network average feature weights onto the published FC probability maps. Primary cortical areas, i.e., sensory-motor, visual, and to some extent, auditory, contribute most to predicting survival

Figure 4 shows the results of the permutation feature importance. Age at diagnosis and sex were both strong predictors of overall survival. CT regions encompassing the superior temporal sulcus, parahippocampal gyrus, pericalcarine, pars triangularis, and middle temporal regions were strong predictors of survival. Supplementary Fig. 5a shows the rank of the CT features based on the anatomical segmentation. Several encoded FC features were also strong predictors. Permutation feature importance on the encoded features (FC10, FC11, and FC4) showed primary involvement with SMDxCON, SMDxVIS, DMNxDMN, SMIxPMN, AUDxPMN, SMIxDMN, AUDxAUD, SMIxVIS, SMDxTHA, and VISxVIS (Supplementary Fig. 5b). When averaging the feature weights for each network, SMD, VIS, CON, and SMI were the strongest average predictors of survival (Fig. 5a). Figure 5b shows the mean and STD of the voxelwise tumor frequency (from Fig. 2) based on RSN segmentations [46]. The networks with the most substantial tumor frequency overlap included AUD, BGA, CON, and SMI. Lastly, Fig. 5c shows the results of mapping the per-network average feature weights onto the published FC probability maps. The associated areas include the motor, occipital, opercular, and anterior insular regions.

## Discussion

The current work demonstrates the utility of machine learning for predicting overall survival in GBM patients. Our results indicate that accurate (> 90%) overall survival classification can be achieved at the time of diagnosis and before treatment using basic demographics (age, sex), cortical thickness, and RS-fMRI connectomics. This is a significant finding considering our patient population varied in tumor location, the extent of surgical resection, treatment course, tumor molecular profiles, medical history, and presenting symptoms (Supplementary Table 1). We assert that our classification of patients into three survival categories can enhance risk stratification and improve shared decision-making between clinicians and individual patients with GBM.

Our model builds upon numerous studies that have demonstrated the ability to predict clinically relevant variables based on MRI, with applications ranging from characterization of intra-tumor genetic heterogeneity [51], to predicting IDH1 [30, 31, 52], PTEN [53, 54], and TERT [54, 55] promoter mutation status and classification of transcriptome-based subtypes (classical, mesenchymal, proneural) [33]. In our past work using RS-fMRI only, we were able to classify long term versus short term survival in GBMs with 72% accuracy [25]. Other studies have successfully classified long versus short term survival with variable cohort sizes and performance [56–59]. Liu et al. trained a support vector machine (SVM) with clinical and FC connectomic features in a cohort of 68 patients with high grade glioma (HGG) to distinguish between survivors with OS < 650 days and those with OS > 650 days with 87% accuracy [59]. Nie et al. used a combination of deep learning and SVMs trained on T1, DTI, and RS-fMRI to classify HGG into similar categories with an accuracy of approximately 91% [60]. These prior studies support our perspective that FC is a potential biomarker for OS. Our ML model was trained on 133 GBM patients, which speaks to the robustness of our findings. Furthermore, we provided detailed clinical contextualization of the comorbidities, clinical presentations, and treatments for each patient. Together these features increase the likelihood that our model will generalize to patients with heterogeneous tumor locations, treatment regimens, and functional statuses.

Our research suggests a notable association between GBMs and alterations in global brain structure and function. These results support our recent findings that cortical thickness and brain-wide BOLD spectra are associated with overall survival and tumor epigenetics [21, 61]. In this current research, we demonstrate that CT in the contralesional superior temporal sulcus, parahippocampal gyrus, pericalcarine, pars triangularis, and middle temporal regions are strong predictors of overall survival (Fig. 4 and Supplementary Fig. 5a). Thus, structural alterations in GBM are not restricted to the immediate vicinity of the tumor. In prior studies, we also observed this phenomenon when comparing contralesional CT in tumor patients with healthy controls [21]. The mechanism underlying contralateral cortical thinning remains unclear. However several intriguing hypotheses have been suggested [21]. First, the “oncologic-metabolic hypothesis” posits that a growing tumor parasitizes nutrients which results in cortical atrophy by depriving other regions of the brain. The more perturbed the global metabolic change, the more aggressive the tumor. Second, a “functional hypothesis” which asserts a locally destructive tumor causes distant effects through altered synaptic homeostasis and diminution of trophic input to remote cortical sites. Third, the “predisposition hypothesis,” which asserts that cortical changes noted at the time of GBMs diagnosis precede oncogenesis and reflect brain health in a patient predisposed to developing a tumor. Alternatively, brain-wide changes could be indicators of global brain health. Although more work is needed to verify our empiric findings, the present results suggest that cortical thickness is an important imaging metric in prediction of overall survival.

We observed that GBMs are associated with widespread changes in resting state networks. The vision network, for example, had the second-strongest average feature weight (Fig. 5a). However, the frequency with which tumors occurred in the occipital lobe in our data set is extremely low (Figs. 2 and 5b). This may seem counterintuitive, but numerous other studies have observed distributed functional alterations [56, 62, 63]. These results support our findings of the prognostic value of functional connectivity in the motor and visual networks. Further, distributed functional connectivity abnormalities in the brain are known to associate with tumor biology and neurocognitive deficits [64, 65]. One possible mechanism of remote functional alteration is inter and intra-network disconnection caused by the lesion on white matter pathways connecting different network nodes [66].

Regions associated with motor function were particularly strong predictors of overall survival. This was primarily observed in the FC analysis, with SMD and SMI showing strong feature weights (Fig. 5a and Supplementary Fig. 5b). Other studies have reported similar results concerning solid tumor connectivity to the frontal lobes and homotopic connectivity of somatomotor networks [57, 58]. The CON, thought to be related to “tonic alertness” (the ability to maintain arousal levels) [67], was also a strong predictor of overall survival. Motor regions were also significant structural features in our model. The contralesional CT analysis revealed moderate predictor strength in the paracentral and precentral gyrus, both associated with motor function [68, 69]. Both the somatomotor and portions of the CON networks are known to be connected and highly involved in activities of daily living, hence, quality of life. This further reinforces the notion that postsurgical functional status is vital to overall survival.

The application of ML to provide prognostic information for patients diagnosed with GBMs preoperatively could impact clinical care in several ways. First, physicians can tailor treatment plans to an individual patient’s life goals and priorities by providing an accurate prognosis. Patients and their families can be provided with realistic expectations regarding the likely course of the disease, survival rates, quality of life, and potential side effects of treatments. This transparency can assist in making informed decisions about treatment options and end-of-life care if applicable. Second, accurate prognostic information can better stratify patients who might benefit from experimental therapies or clinical trials. This can facilitate faster development of new treatments for GBM. Third, in healthcare systems with limited resources, prognostic algorithms may assist in prioritizing patients who may benefit most from aggressive interventions and specialized care while also considering cost-effective palliative care for those with a less favorable prognosis.

This work has several limitations. First, our data were collected at a single institution. Future work will involve further model validation at multiple sites and prospective validation at our institution. Future work should also involve a similar analysis using progression-free survival as the outcome measure and compare those results with the current work. Further, although we provided statistics on tumor location, genetic alterations, medical history, and presentation symptoms, these variables were not included in our models. This was by design, as we wanted to assess predictive ability based on neuroimaging data and minimize the use of demographics. Still, future work should consider including additional variables to ensure stable model performance. Also, future work should consider combining ML’s pattern discernment and predictive ability with traditional statistical approaches such as intent-to-treat analysis and Cox proportional hazards models. Such combined analyses could reveal nuanced interactions in the data and offer a more holistic understanding of survival predictions, addressing both the micro-level intricacies captured by ML and the macro-level statistical relationships. Lastly, a central goal of future work is to extend beyond the academic realm by actively deploying and integrating our models into the clinical setting. This ensures that GBM patients directly benefit from these advanced analytical tools.

## Conclusion

In this research, we have demonstrated the ability of machine learning to accurately classify overall survival in GBM patients before any treatment (including surgery or chemoradiation) using age, sex, cortical thickness, and resting state functional connectivity. Our results suggest that GBMs are associated with global structural and functional alterations in the brain, extending past the tumor’s location. We assert that these models can potentially improve patient care by facilitating individualized treatment plans, informing clinical trial enrollment at the earliest possible stage, and shared decision-making.

### Electronic supplementary material

Below is the link to the electronic supplementary material.

Supplementary Material 1

Supplementary Material 2

## Data Availability

The data used in this study will be made available after approval from the appropriate study PIs (Eric Leuthardt, Joshua Shimony).
