# Supplementary material for: Predicting survival in glioblastoma with multimodal neuroimaging and machine learning
Source: J Neurooncol. 2023 Sep 5;164(2):309–20. doi: 10.1007/s11060-023-04439-8 (PMC10522528; doi:10.1007/s11060-023-04439-8)
Supplement: Supplementary file 1 — Supplementary Material 1 [file 11060_2023_4439_MOESM1_ESM.pdf]

Predicting Survival in Glioblastoma with Multimodal Neuroimaging and Machine Learning
Journal of Neuro-Oncology
Luckett et al.
Luckett.patrick@wustl.edu

1.1 MRI Processing

Preprocessing of fMRI data followed previously described methods, including compensation for slice-dependent time shifts, elimination of systematic odd-even slice intensities, and rigid body correction of head movement [1–3]. The data were then resampled in 3mm^3 atlas space using affine transformations to the T1w structural image. Further processing included voxel-wise removal of linear trends, temporal low-pass filtering retaining frequencies below 0.1Hz, regression of nuisance waveforms, and spatial smoothing with 6mm full width at half maximum Gaussian blurring in each direction. Frame censoring was implemented using the DVARS measure as previously described [1].

1.2 Tumor Segmentation

Automated tumor segmentation was performed with a pre-trained convolutional neural network architecture [4] using post-contrast T1w, T2w, and FLAIR scans. The algorithm segments vasogenic edema, necrotic/non-enhancing core, and enhancing core. Tumor segmentations were visually examined and edited using ITK-SNAP to ensure precise delineation. Active contour techniques were utilized in ITK-SNAP, with reference to T1w, T2w, and FLAIR scans, to generate a semi-automatic tumor segmentation. Furthermore, manual segmentation was used to approximate areas with interrupted contours. Inadequate segmentations resulted in the exclusion of the sample. A whole tumor mask, including all three tissue classes, was used for masking during the atlas registration of the structural and functional images. Further, the automated tumor segmentation maps were used to create voxelwise heat maps, which showed the relevant frequency of a voxel overlapping with the tumor segmentation. For each map, regions not contained in the segmentation were assigned a value of zero, areas segmented as edema were assigned a value of 1, and the non-enhancing/enhancing core was assigned a value of 2. These segmentations were then averaged to generate voxel-wise heat maps of tumor frequency in atlas space.

1.3 Machine Learning and Statistical Analysis

Survival prediction was achieved using deep feedforward artificial neural networks (ANN). The ANNs used in our analysis consisted of 3 hidden layers with eight neurons in each layer (Supplemental Figure 1A). Each neuron utilized a normalized radial basis transfer function. The model was trained to classify patients into less than one year, between one and two years, or greater than two years of survival. Initially, multiple network architectures were trained and compared, with the smallest network containing a single hidden layer and five artificial neurons and the largest network containing five hidden layers with 20 artificial neurons in each layer. The network with three hidden layers and eight neurons showed the best performance.

Input to the ANNs included age, sex, contralesional CT measures [5], and FC features. Due to many FC features (120) versus the size of our data set, dimensionality reduction was performed using an autoencoder. Autoencoders are a type of feedforward neural network that attempts to map an input back to itself [6]. In between the input and output layer exist a single hidden layer that encodes the original input into a reduced dimension feature space (based on the number of neurons in the hidden layer), which is then decoded back to an approximation of the original data. Supplemental Figure 1B depicts the autoencoder used in our analysis. The autoencoder consisted of a single hidden layer with 20 neurons with normalized radial basis transfer functions. A total of 11 encoded FC features (after removing nine sparse features) were used for training the model and are hereafter referred to as FC1, FC2,…FC11.

Supplemental Figure 2 describes the survival model training and validation procedure. The initial data set comprised 133 samples; 10 stratified samples were held out, which were not used for training. Then, the data was partitioned into 26 folds. Each fold held out approximately five stratified samples not used for training to serve as within-fold validation testing. Then within each fold, a model was trained using 70% of the training data of the given fold. The remaining 30% of training data was used for model validation termination, such that the model stopped training once the performance on the 30% validation data did not improve after ten epochs. Once the models finished training, they were tested on their corresponding within-fold validation data and the original ten held-out samples. The autoencoder was trained on 80% of the data, with 20% reserved for validation termination.

Permutation feature importance [7] was used to identify the strongest predictive features of survival based on the trained models. Permutation feature importance involves evaluating a trained model's performance on a given data set. Once the model performance is established, a single feature is randomly permuted in the original data set, and the data is tested on the model. After the given feature is permuted, the model performance is noted, and the process is repeated for each successive feature. The influence of a given feature is based on the impact that permuted feature had on the accuracy of the model compared to the accuracy of the model with no permuted features. Thus, features that significantly impact model performance are considered strong predictors. This process was conducted for each model trained in the cross-validation folds and evaluated based on the global hold-out data and the data held out for the given fold. The process was repeated 1000 times for each model, and all results were combined and averaged to get a single weight for each feature. The feature weights were first identified for the CT and the autoencoded FC features based on the survival prediction models. Once the feature weights were established for the autoencoded FC features, the process was repeated on the autoencoder. The performance measure we evaluated was based on the impact of permuting a given feature on the autoencoded FC features in question. This allowed identifying the strongest within and between network FC features without directly using those features in the model. Further, an average per-network feature weight was generated by averaging all within and between-network feature weights for each given network (e.g., to calculate the average feature weight for SMD, we averaged all feature weights associated with SMD [SMDxSMD, SMDxSMI, SMDxCON…..]). Lastly, voxelwise FC feature maps were calculated by taking the dot product of the average feature weights with publically available FC probability maps [8]. The FC probability maps were generated using a deep learning algorithm, and results were averaged over ~2000 participants. Each voxel in the probability maps consists of a softmax probability distribution representing the probability of that voxel belonging to each of the given networks. Thus summing the multiplication of the mean feature weights with the associated voxelwise softmax probabilities yields an overall feature weight for each voxel. The survival prediction pipeline and methods described herein are summarized in Figure 1 and detailed in the supplementary figures.

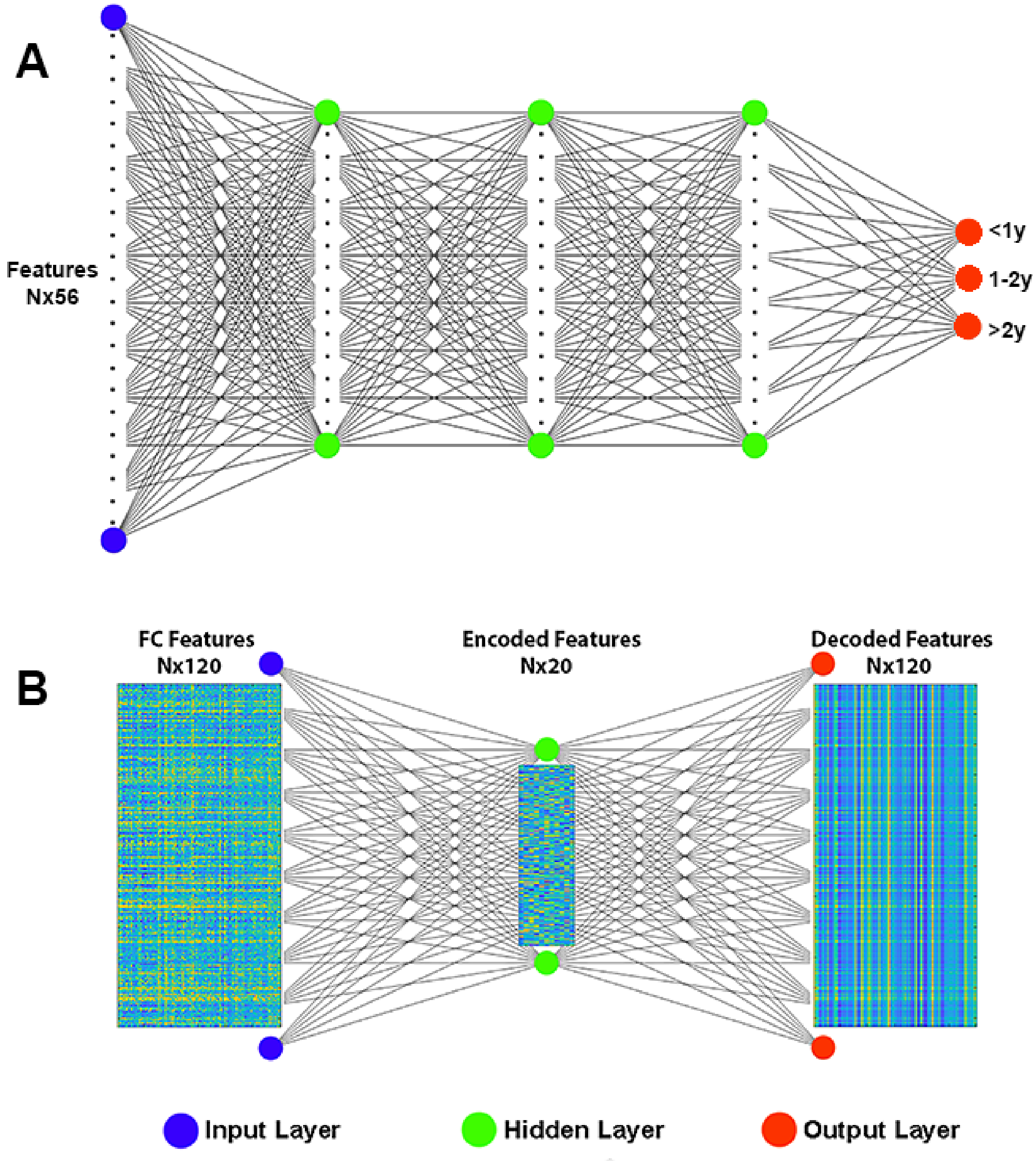

Supplementary Fig 1: A) ANN architecture used for classifying patients into survival category (<1y, 1-2y, >2y) consisting of 3 hidden layers, eight neurons in each layer, and normalized radial basis transfer functions. B) Autoencoder architecture used for reducing the dimension of FC features consisting of a single hidden layer, 20 neurons, and normalized radial basis transfer functions.

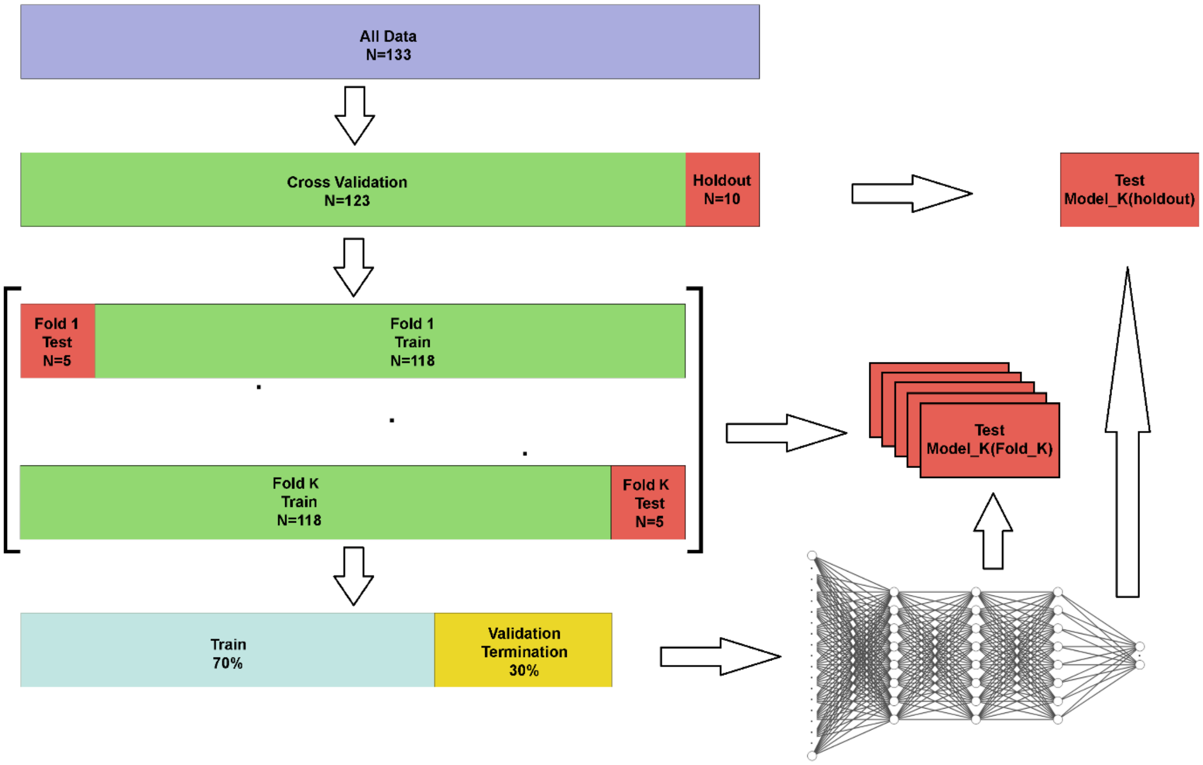

Supplementary Fig 2: Survival model validation procedure. The initial data set was composed of 133 samples. Ten stratified samples were held out, which were not used for training. The data was partitioned into 26 folds, each holding out approximately five stratified samples not used for training in the given fold. Within each fold, a model was trained using 70% of the training data of the given fold. The remaining 30% of training data was used for model validation termination. After training, the models were tested on their corresponding within-fold validation data, as well as the original ten held out samples that were not trained on by any model.

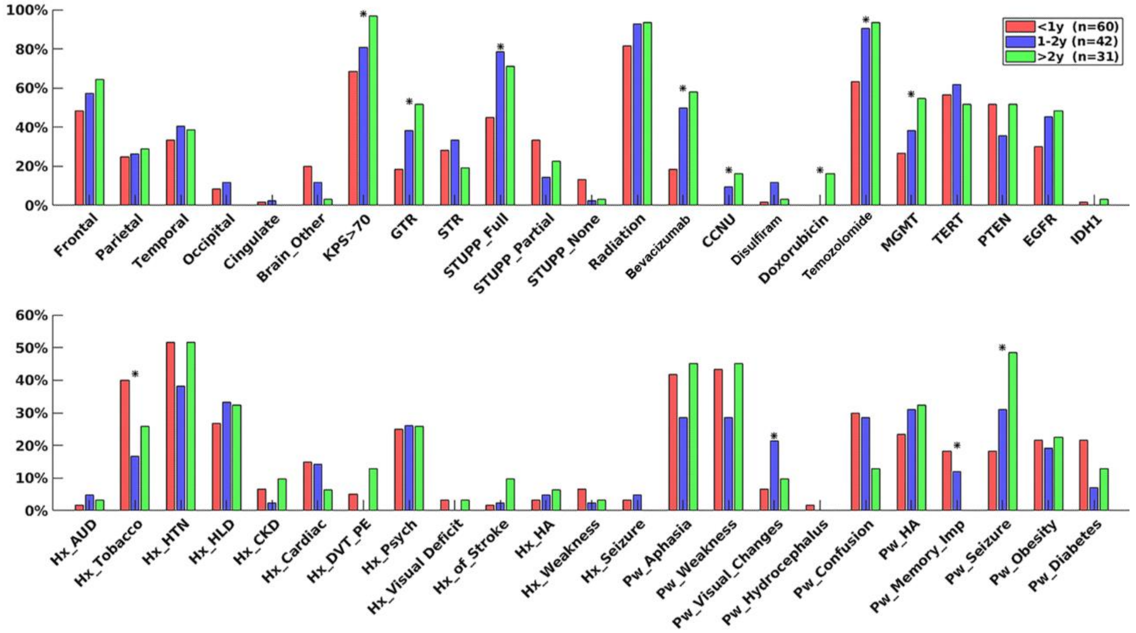

Supplementary Fig. 3: Ratios of variables based on survival category. KPS>70, gross total resection (GTR), full STUPP protocol, bevacizumab, CCNU, doxorubicin treatment, temozolomide treatment, MGMT promoter methylation status, history of tobacco use, presenting with visual changes, presenting with memory impairment, and presenting with seizures showed significant differences between the three survival groups (Chi-squared test).

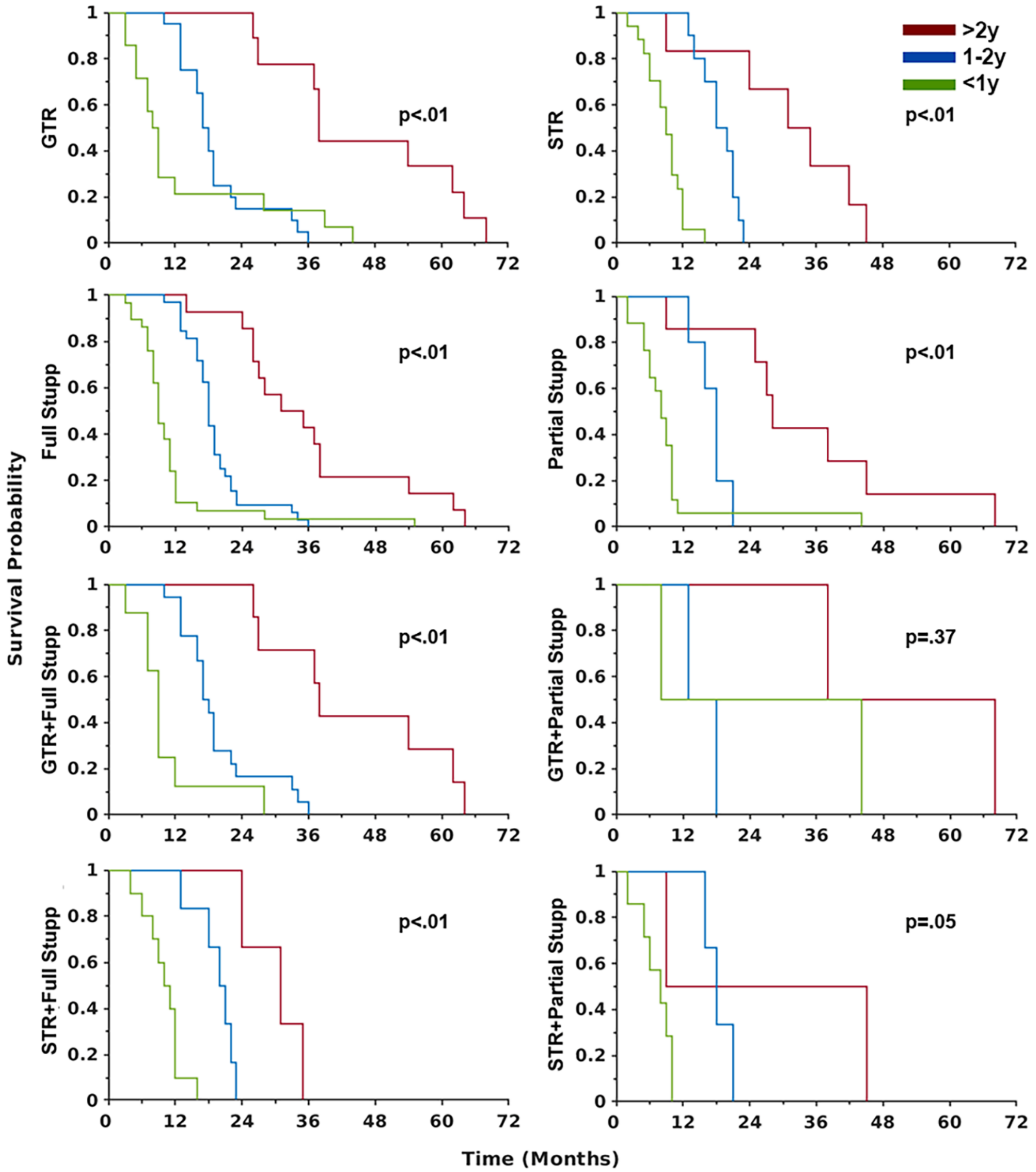

Supplementary Fig 4 Kaplan-Meier survival curves were generated based on model classification, partitioned by extent of resection (gross total (GTR) or subtotal (STR)), completion of full or partial Stupp protocol, and the intersection of these measures. P-values were significant (p<.01) for all factors, with the exception of GTR and STR combined with a partial Stupp protocol. This suggests that the model can accurately classify survival in GBM patients prior to surgical, chemical, or radiotherapy treatments.

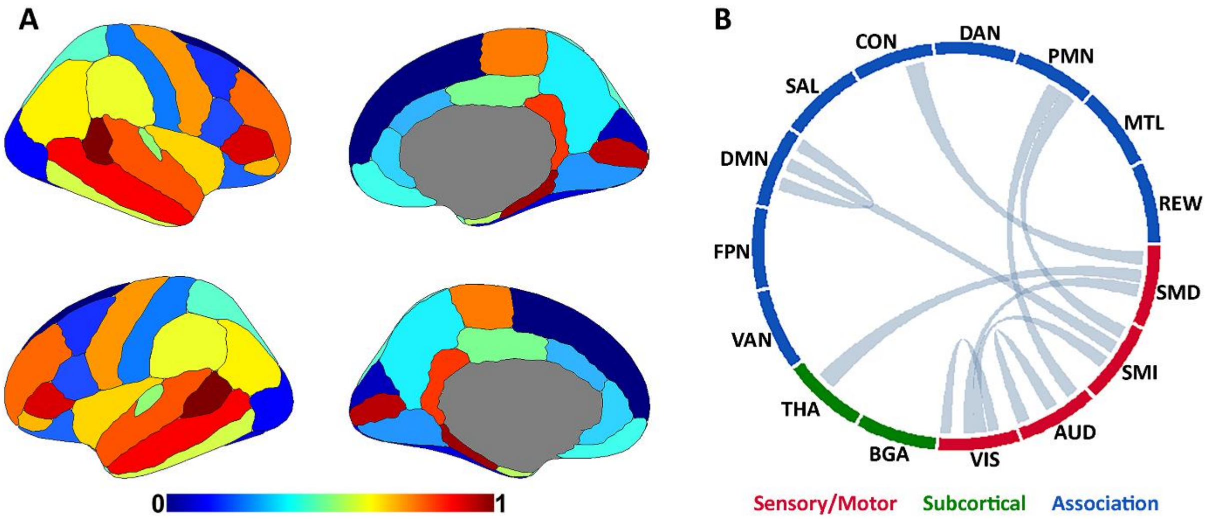

Supplementary Fig. 5 A) Strongest predictive cortical thickness features of survival. The strongest predictors included banks of the superior temporal sulcus, parahippocampal gyrus, pericalcarine, pars triangularis, and middle temporal regions. B) Top 10 FC features derived from permutation feature importance of the top encoded FC features (FC10, FC11, and FC4 (Figure 4). SMDxCON, SMDxVIS, DMNxDMN, SMIxPMN, AUDxPMN, SMIxDMN, AUDxAUD, SMIxVIS, SMDxTHA, and VISxVIS were the strongest features.

1. Power JD, Barnes KA, Snyder AZ, et al (2012) Spurious but systematic correlations in functional connectivity MRI networks arise from subject motion. Neuroimage. https://doi.org/10.1016/j.neuroimage.2011.10.018
2. Shulman GL, Pope DLW, Astafiev S V, et al (2010) Right hemisphere dominance during spatial selective attention and target detection occurs outside the dorsal frontoparietal network. J Neurosci 30:3640–3651
3. Park KY, Shimony JS, Chakrabarty S, et al (2023) Optimal Atlas Registration and Resting State Functional Architecture in Patients with Glioblastoma. NeuroImage Clin
4. Isensee F, Kickingereder P, Wick W, et al (2017) Brain tumor segmentation and radiomics survival prediction: Contribution to the brats 2017 challenge. In: International MICCAI Brainlesion Workshop. Springer, pp 287–297
5. Lamichhane B, Luckett PH, Dierker D, et al (2023) Structural Gray Matter Alterations in Glioblastoma and High Grade Glioma-A Potential Biomarker of Survival. Neuro-Oncology Adv vdad034
6. Goodfellow I, Bengio Y, Courville A (2016) Deep Learning. MIT Press
7. Fisher A, Rudin C, Dominici F (2019) All Models are Wrong, but Many are Useful: Learning a Variable's Importance by Studying an Entire Class of Prediction Models Simultaneously. J Mach Learn Res 20:1–81
8. Luckett PH, Lee JJ, Park KY, et al (2022) Resting state network mapping in individuals using deep learning. Front Neurol 13:
