# Supplementary material for: Predicting survival in glioblastoma with multimodal neuroimaging and machine learning
Source: J Neurooncol. 2023 Sep 5;164(2):309–20. doi: 10.1007/s11060-023-04439-8 (PMC10522528; doi:10.1007/s11060-023-04439-8)
Supplement: Supplementary file 2 — Supplementary Material 2 [file 11060_2023_4439_MOESM2_ESM.pdf]

Predicting Survival in Glioblastoma with Multimodal Neuroimaging and Machine Learning
Journal of Neuro-Oncology
Luckett et al.
Luckett.patrick@wustl.edu

	All Data	<1y	1-2y	>2y	p-value
N	133	60	42	31	
Survival(m) (Median±MAD)	14.1±9.9	7.6±3.1	17.4±2.9	37.6±12.4	<.01
Age(y) at Diagnosis (Mean±STD)	60.8±12.0	63.9±10.8	58.7±11.3	57.8±14.1	0.04
Sex (Male)	57.9%	56.7%	42.9%	80.1%	0.01
KPS>70	78.9%	68.3%	81%	96.8%	0.01
Tumor Location					
Frontal Tumor	54.9%	48.3%	57.1%	64.5%	0.32
Parietal Tumor	26.3%	25%	26.2%	29%	0.92
Temporal Tumor	36.8%	33.3%	40.5%	38.7%	0.74
Occipital Tumor	7.5%	8.3%	11.9%	0%	0.15
Cingulate Tumor	1.5%	1.7%	2.4%	0%	0.7
Other Tumor	13.5%	20%	11.9%	3.2%	0.08
Treatment Protocols					
GTR	32.3%	18.3%	38.1%	51.6%	<.01
STR	27.8%	28.3%	33.3%	19.4%	0.42
Full STUPP	61.7%	45%	78.6%	71%	<.01
Partial STUPP	24.8%	33.3%	14.3%	22.6%	0.09
No STUPP	7.5%	13.3%	2.4%	3.2%	0.07
Radiation	88%	81.7%	92.9%	93.5%	0.13
Bevacizumab	37.6%	18.3%	50%	58.1%	<.01
CCNU	6.8%	0%	9.5%	16.1%	0.01
Disulfiram	5.3%	1.7%	11.9%	3.2%	0.06
Doxorubicin	3.8%	0%	0%	16.1%	<.01
Temozolomide	78.9%	63.3%	90.5%	93.5%	<.01
Molecular Features					
MGMT	36.8%	26.7%	38.1%	54.8%	0.03
TERT	57.1%	56.7%	61.9%	51.6%	0.68
PTEN	46.6%	51.7%	35.7%	51.6%	0.23
EGFR	39.1%	30%	45.2%	48.4%	0.14
IDH1	1.5%	1.7%	0%	3.2%	0.53
Patient History					
Hx Alcohol use disorder	3%	1.7%	4.8%	3.2%	0.66
Hx Tobacco	29.3%	40%	16.7%	25.8%	0.03
Hx Hypertension	47.4%	51.7%	38.1%	51.6%	0.35
Hx Hyperlipidemia	30.1%	26.7%	33.3%	32.3%	0.74
Hx CKD	6%	6.7%	2.4%	9.7%	0.41
Hx Cardiac	12.8%	15%	14.3%	6.5%	0.48
Hx DVT/PE	5.3%	5%	0%	12.9%	0.05
Hx Psychiatric disorders	25.6%	25%	26.2%	25.8%	0.81

Hx Visual Deficit	2.3%	3.3%	0%	3.2%	0.49
Hx Stroke	3.8%	1.7%	2.4%	9.7%	0.14
Hx Headache	4.5%	3.3%	4.8%	6.5%	0.79
Hx Weakness	4.5%	6.7%	2.4%	3.2%	0.55
Hx Seizure	3%	3.3%	4.8%	0%	0.49
Hx Obesity	21.1%	21.7%	19%	22.6%	0.92
Hx Diabetes	15%	21.7%	7.1%	12.9%	0.12
Presentation Symptoms					
Pw Aphasia	38.3%	41.7%	28.6%	45.2%	0.27
Pw Weakness	39.1%	43.3%	28.6%	45.2%	0.24
Pw Visual Changes	12%	6.7%	21.4%	9.7%	0.02
Pw Hydrocephalus	0.8%	1.7%	0%	0%	0.54
Pw Confusion	25.6%	30%	28.6%	12.9%	0.18
Pw Headache	27.8%	23.3%	31%	32.3%	0.4
Pw Memory Impairment	12%	18.3%	11.9%	0%	0.04
Pw Seizure	29.3%	18.3%	31%	48.4%	0.01

Supplementary Table 1: Patient Demographics. Continuous data used the Kruskal-Wallis test, ratios used chi squared test. Hx = History of, Pw = presented with, STD = standard deviation, MAD = median absolute deviation
